# Impact of the COVID-19 pandemic on initiation of antihypertensive drugs in Sweden: an interrupted time series study

**DOI:** 10.1136/bmjopen-2023-082209

**Published:** 2024-10-16

**Authors:** Ana Tomas, Bjorn Wettermark, Fredrik Nyberg, Mohammadhossein Hajiebrahimi

**Affiliations:** 1 Department of Pharmacology and Toxicology, Faculty of Medicine Novi Sad, University of Novi Sad, Novi Sad, Serbia; 2 Pharmacoepidemiology & Social Pharmacy, Department of Pharmacy, Uppsala University, Uppsala, Sweden; 3 School of Public Health and Community Medicine, Institute of Medicine, Sahlgrenska Academy, University of Gothenburg, Goteborg, Sweden

**Keywords:** COVID-19, EPIDEMIOLOGIC STUDIES, Hypertension

## Abstract

**Abstract:**

**Objectives:**

Antihypertensives reduce the risk of myocardial infarction and stroke. Restrictions during the COVID-19 pandemic limited access to healthcare, which may have had a negative impact on drug prescribing. This study aimed to assess the effect of the COVID-19 pandemic on the initiation of antihypertensive drugs.

**Design:**

Interrupted time series study using a segmented linear regression model.

**Setting:**

Swedish population assessed through linked national healthcare registers.

**Participants:**

720 300 new users of antihypertensives.

**Intervention:**

March 2020, COVID-19 pandemic onset.

**Main outcomes measures:**

The change in the initiation of antihypertensives expressed as monthly cumulative incidence, stratified by age and sex. Data on dispensed prescriptions of diuretics, beta-blockers, calcium channel blockers, ACE inhibitors (ACEi) and angiotensin receptor blockers were extracted from the Swedish Prescribed Drug Register, from March 2018 to November 2021. Initiation (new use) was defined as having no previous dispensations before March 2019. Monthly cumulative incidence in March 2019–November 2021 was calculated as the number of patients initiating each drug class in each month divided by the population.

**Results:**

The start of the pandemic was associated with an immediate drop in the initiation of any antihypertensive, but no sustained effects were observed, as the incidence continued to increase in the postinterruption period by +0.02% each month in both sexes. The immediate drop was statistically significant for ACEi in both sexes and all antihypertensive classes except diuretics in patients >65 years. А significant postintervention trend change was observed for initiation of diuretics (+0.013% overall), driven mainly by a significant increase in patients >65 years. Similar findings were also observed for diuretics in females (+0.02%) and ACEi (+0.03%) in patients >65 years.

**Conclusions:**

The pandemic had an immediate negative short-term effect, but we found no major long-term negative influence of the COVID-19 pandemic on initiation of any type of antihypertensive drugs.

**STRENGTHS AND LIMITATIONS OF THIS STUDY**

- The complete national coverage of all antihypertensive drugs dispensed in Sweden, integrated with data from other health registries, constitutes an important strength of this study.
- The Swedish Prescribed Drug Register does not include information about diagnoses/reasons for prescribing, so we were unable to assess if the patients initiating antihypertensives had hypertension or other underlying diseases such as heart failure or ischaemic heart disease.
- Furthermore, all data represent dispensed prescriptions and with no information on prescriptions issued but not filled at pharmacies.
- Due to the observational study design, we can only infer a causal correlation between the COVID-19 pandemic onset and the studied outcomes, and we cannot exclude the possibility of other coinciding factors driving the observed changes.

## Introduction

 In March 2020, COVID-19 was declared a global pandemic by the WHO, just months after the first case of infection with novel SARS-CoV-2 was confirmed in Wuhan, China.^1^ The pandemic caught the world unprepared, causing an unprecedented impact in the contemporary era, with more than 287 million confirmed cases and 5.4 million deaths directly attributed to the virus and an estimated 15 million excess deaths, in 2020 and 2021 alone.^2^ The pandemic has challenged health systems globally, with resources and attention diverted to address the immediate and overwhelming demands. COVID-19 caused widespread disruptions in health services that affected the care of patients with chronic conditions.^3 4^ Such disruptions are particularly concerning for cardiovascular diseases (CVDs), due to their high morbidity and mortality,^5 6^ but also complicated interplay between CVD and COVID-19.

CVD is a risk factor for severe COVID-19, but the COVID-19 infection itself also negatively affects CVD outcomes, with a substantial burden of CVD in COVID-19 survivors.^7^,^9^ Patients with pre-existing CVD were found to be more susceptible to COVID-19 infection while also having an increased risk of severe COVID-19 disease, hospitalisation and death,^10^,^12^ with hypertension identified as the most common coexisting condition in patients infected with COVID-19.^8 13^ On the other hand, COVID-19 is associated with several direct and indirect cardiovascular complications, including microvascular disease, acute myocardial injury, myocarditis, cardiomyopathies, cardiac dysfunction, arrhythmias and venous thromboembolism.^10 14^ CVD risk factors, such as hyperlipidaemia, hypertension, diabetes and obesity, were also found to be associated with worse COVID-19 outcomes.^12^ What is more, studies on the impact of COVID-19 pandemic on CVD burden reported a reduction in rates of cardiovascular-related and cerebrovascular-related referrals, diagnoses and treatments during the pandemic compared with the prepandemic period.^15^,^18^ A large Dutch, nationwide cohort study found a decline in incidence rates of in-hospital diagnosis of ischaemic stroke, myocardial infarction and other cardiovascular conditions during the first wave of the pandemic, with rates returning to prepandemic levels during 2020. Possible reasons for this decline include patients avoiding or delaying care, hesitancy to refer patients to the emergency departments and the competing risk of dying from COVID-19. Potentially missed diagnoses of CVD can lead to health complications and excess mortality, both due to lack of acute treatment and consequent secondary preventive care. Therefore, in the COVID-19 era, timely detection and implementation of optimal therapeutic strategies for CVD risk factor control is of utmost importance. Antihypertensive drugs, in addition to providing blood pressure control, are known to be effective in lowering the risk of myocardial infarction, heart failure and stroke.^14 19^

Early during the pandemic, controversies arose on the role of antihypertensive agents in the outcomes of COVID-19 infection and the appropriate choice of antihypertensives. Much focus was on drugs affecting the renin–angiotensin–aldosterone system (RAAS), specifically the ACE inhibitors (ACEis) and angiotensin receptor blockers (ARBs), due to the evidence of the SARS-CoV-2 virus requiring ACE2 receptor for the entry into host cells.^20^ As previous studies suggested that ACEis and ARBs can increase ACE2 expression,^16 17 21 22^ an increased susceptibility to and worsened prognosis from COVID-19 in patients treated with RAAS was postulated. Multiple observational studies and clinical trials ensued, and the evidence summarised in several systematic reviews and meta-analyses shows that the use of RAAS inhibitors does not increase the risk of mortality or severity of COVID-19,^23^,^28^ but rather suggests beneficial effects and lower risk of poor outcomes among hypertensive patients already using these drugs.^23 25 29^ An umbrella review of 46 systematic reviews found that the evidence is strong for the continued use of ACEIs/ARBs being associated with reduction in death and ICU admission, supporting continuing ACEIs/ARBs therapy in patients with COVID-19.^20^

For other antihypertensive drug classes, the findings differ. For calcium channel blockers (CCBs), based on their mechanism of action, there were discussions of CCBs worsening ventilation/perfusion mismatch, further exacerbating hypoxaemia,^30^ but also CCBs facilitating oxygen delivery in COVID-19 patients.^31^ Recent meta-analyses revealed a significant reduction in the odds of all-cause mortality with prior use of CCBs relative to the non-use of CCBs in hypertensive patients with COVID-19.^32 33^ However, there was no evidence of an association between prior CCB use and COVID-19 incidence and severity.^29^ Regarding diuretics and beta blockers (BBs), there is less evidence of the role in COVID-19 susceptibility, morbidity and mortality. A meta-analysis suggested no association between the use of BBs and diuretics with incidence or severity of COVID-19.^29^ Nonetheless, in general, the evidence clearly favours continued treatment with antihypertensives during the pandemic, but all these uncertainties imply the further need to identify the impact the pandemic has had on the antihypertensive drug use.

A Swedish cohort study found that all antihypertensives were initiated more frequently in COVID-19 patients compared with a matched group from the general population.^34^ Possible explanations included onset of hypertension due to the COVID-19 infection, more frequent detection of hypertension diagnosis in COVID‐19 patients or some residual confounding factors, for example, people having high body mass index or other risk factors facing both a higher risk for COVID-19 and hypertension, respectively.^34^

How the pandemic has affected the treatment of antihypertensives is scarcely described in the literature. Learning from the pandemic is important to prepare better for future crises. Taking all of the above into account, this study aimed to assess the effect of the COVID-19 pandemic on the initiation of antihypertensive drugs.

## Material and methods

### Setting

This population-based interrupted time series analysis of dispensed antihypertensive drugs was conducted in Sweden. Sweden has a population of approximately 10.4 million inhabitants, with universal, mainly government-funded, decentralised healthcare organised in 21 regions. Around 11% of the gross domestic product (GDP) is annually invested in healthcare, with high public financing (86%) and public provision (83%). Voluntary health insurance represents less than 1% of total health expenditures.^35^ The study was conducted as part of the Swedish Covid-19 Investigation for Future Insights—a Population Epidemiology Approach using Register Linkage (SCIFI-PEARL) project, with a study period from March 2018 to November 2021. All patients with at least one dispensed prescription of an antihypertensive drug during the study period were included in the population.

### Data sources

Data were obtained from the SCIFI-PEARL project dataset.^36^ SCIFI-PEARL is a linked multiregister project combining high-quality national registers with continuously repeated linkage and regular updates, successively expanded, now covering the whole Swedish population. SCIFI-PEARL also identifies all PCR test positive COVID-19 patients and links the dataset to other Swedish health registers at individual level using unique personal identification numbers, in order to identify covariates and outcomes.^37^ Data on monthly population estimates stratified based on age and sex originate from Statistics Sweden.^38^ Data on dispensed prescription drugs from 1 January 2018 come from the Swedish National Prescribed Drug Register.^39^

### Intervention

The intervention point was defined as the start of the COVID-19 pandemic in March 2020. The period between March 2019 and February 2020 was considered as preintervention period and April 2020–November 2021 as the postintervention period, due to the washout period required (see below) and drug register data availability only from 2018. On 11 March 2020, COVID-19 was declared a global pandemic by the WHO. Several measures were introduced during the pandemic to limit the infection spread and keep critical services operational. In Sweden, a somewhat different strategy was chosen to control the pandemic, with no general lock-downs, use of recommendations focusing more on slowing, rather than stopping, the pandemic and measures introduced to ensure a functioning healthcare system.^40 41^ These were a combination of binding regulations and voluntary measures, aiming to reduce the pressure on and provide continuity of health and medical care services. They included increased allocation of funds to the healthcare system due to increased demands and costs during the pandemic, measures to ensure medicine availability and limit shortages, and support of remote visits to healthcare providers.^42^

### Outcome

The main outcome of interest was the initiation of antihypertensive drugs, expressed as monthly cumulative incidence. Initiation was defined as first dispensing (new use) after a 1-year drug-free washout period and calculated by dividing the number of individuals who dispensed their first prescription each month by the corresponding monthly population, stratified by age and sex. A fixed washout period, between January 2018 and March 2019 was used for all participants. The patients who initiated their antihypertensive drugs later, thus practically had a longer washout period. Initiation was determined in the period between March 2019 and November 2021. The following drug classes according to the Anatomic Therapeutic Chemical classification system codes were assessed: diuretics (C03), BBs (C07), CCBs (C08, including C07FB02), ACEis (C09A), ARBs (ARB; C09C), and fixed combinations of ACEis/thiazides (C09B) and ARBs/thiazides (C09D) were included in the study. Variables used to describe the population were age at first dispensing (calculated by subtracting birth date from the dispensing date), categorised into two groups for the interrupted time series analysis (18–64 and >65 years) and sex (male/female).

### Statistical analysis

The number of individuals initiating antihypertensive drugs each month, divided by the monthly population, was determined, and the single interrupted time-series analysis (SITSA) using a segmented linear regression model was used to assess whether the cumulative monthly incidence of initiations on antihypertensive drugs changed in response to the COVID-19 pandemic. 12 time points before the pandemic and 20 time points after the start of the pandemic were included. Detailed explanations about SITSA model and its context are available elsewhere^43^ but in brief, the equation of the model was as follows: y=α+β1T+β2X+β3XT+ε where y=outcome variable, α=intercept, β=coefficients (β1=preintervention trend (%), β2=level change following the intervention (%), β3=postintervention slope (unit), β1+β3=postintervention trend (%)), T=time, X=study phase, XT=time after the interruption and ε=error or residual term. Residuals were assessed for normality of distribution. ITS results were plotted to include data points for monthly incidence; the interruption point in March 2020; preinterruption and postinterruption trend lines; and the counterfactual postinterruption trend line.^44^ All analyses were conducted by using SAS V.9.4 (SAS Institute); with statistical significance assessed at a level of 5% and 95% CIs calculated for point estimates.

### Patient and public involvement

Patients or the public were not involved in this study, which is based on secondary data.

## Results

Among 720 300 individuals who initiated antihypertensives between March 2019 and November 2021, 381 881 (53.0%) were female, with an average age of 60.0±16.8 years (table 1). Characteristics of patients initiating different drug classes are presented in online supplemental table 1.

**Table 1:** Characteristics of the study population initiated on antihypertensive drugs in Sweden between March 2019 and November 2021

Variables	Numbers	Per cent
Total	720 300	
Sex		
Male	338 419	47.0
Female	381 881	53.0
Year of first dispensing		
2019	266 527	37.0
2020	245 921	34.1
2021	207 852	28.9
Age		
<18	4836	0.7
18–64	402 314	55.9
≥65	313 150	43.5
Marital status		
Married	339 700	47.2
Unmarried	379 098	52.6
Missing*	1502	0.2
Educational level		
Primary school	151 662	21.1
Secondary school	317 845	44.1
Tertiary eductaion	232 934	32.3
Unknown	17 859	2.5
Occupation status		
Employed	422 994	58.7
Unemployed/retired	290 814	40.4
Missing*	6492	0.9
Country of birth		
Sweden	578 476	80.3
Nordics excluding Sweden	27 677	3.8
Eu28 except the Nordics	27 341	3.8
Out of Europe	86 762	12.1
Missing*	44	0.0

* Individuals with data unavailable.

**Figure 1:**
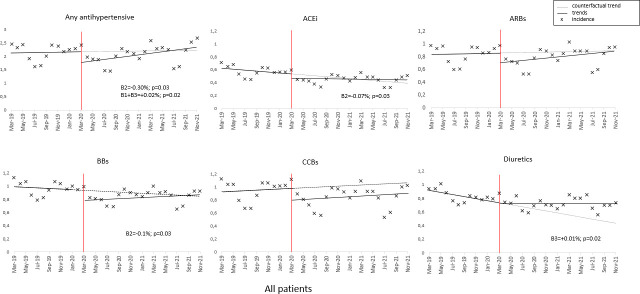
Cumulative monthly incidence of initiation of antihypertensives drugs, Sweden, March 2019–November 2021. Red line: interruption point (the start of the pandemic), full lines: preinterruption and postinterruption trends, dotted line: counterfactual trend. Parameters: β0: intercept value; β1: pretrend slope; β2: postinterruption immediate level change; β3: postinterruption trend change; β1+β3: postinterruption trend slope. Parameters with probability <0.05 are presented. ACEi, ACE inhibitors; ARBs, angiotensin receptor blockers; BBs, beta blockers; CCBs, calcium channel blockers.

Monthly cumulative incidences per 1000 inhabitants for initiation of the major drug classes ACEIs, ARBs, BBs, CCBs and diuretics are shown in figure 1. From March 2020 to February 2021, for each month, the cumulative incidence was lower compared with the same months of 2019. From March 2021, higher values were observed for June 2021 compared with June 2019, and from September to November 2021 compared with the same months of 2019 (online supplemental table 2). The results of SITSA, analysing if postinterruption time points differ statistically significantly from what would have occurred in the absence of an interruption, estimated a non-significant (p=0.88) prepandemic trend with rates increasing by only +0.0028% per month (β_1_). After controlling for this trend, there was a significant (p=0.03) level change (β_2_) at the interruption time point, indicating an abrupt reduction in the initiation of any antihypertensive initiation. The postinterruption trend did not significantly differ from the prepandemic period (β3=+0.016%, p=0.46), but the postinterruption slope as such was significant (p=0.016) with rates continuing to increase by +0.019% per month (β_1_+β_3_). Similar findings were observed after sex stratification, with a significant level change (−0.30% and −0.31% for females and males, respectively) (table 2, online supplemental figures 1 and 2). A significant postinterruption slope was found for both sexes and all age groups (table 3, online supplemental figures 3 and 4), with the biggest increase observed for older than 65, with a +0.06% monthly increase for the postintervention period.

**Table 2:** Single interrupted time-series analysis for cumulative incidence of any antihypertensive, ACEi, ARBs, BBs, CCBs and diuretics initiation before and after the pandemic onset by sex in Sweden

Drug	Parameter	Male			Female		
		Coefficient (%)	95% CI	P value	Coefficient (%)	95% CI	P value
Any	β0	1.973	1.699 to 2.247	<0.0001	2.299	2.020 to 2.578	<0.0001
	β1	0.007	−0.030 to 0.043	0.724	−0.001	−0.037 to 0.036	0.964
	β2	−0.289	−0.542 to −0.037	0.033	−0.313	−0.576 to −0.049	0.027
	β3	0.01	−0.033 to 0.053	0.658	0.024	−0.018 to 0.066	0.277
	β1+β3	0.016	0.001 to 0.032	0.039	0.023	0.007 to 0.038	0.004
ACEi	β0	0.679	0.603 to 0.754	<0.0001	0.583	0.514 to 0.652	<0.0001
	β1	−0.008	−0.018 to 0.001	0.106	−0.006	−0.015 to 0.003	0.237
	β2	−0.074	−0.141 to −0.007	0.039	−0.08	−0.146 to −0.014	0.025
	β3	0.006	−0.005 to 0.017	0.271	0.006	−0.004 to 0.015	0.274
	β1+β3	−0.002	−0.005 to 0.001	0.231	0	−0.003 to 0.003	0.969
ARBs	β0	0.83	0.694 to 0.965	<0.0001	0.84	0.714 to 0.966	<0.0001
	β1	0.004	−0.014 to 0.022	0.694	0	−0.017 to 0.016	0.984
	β2	−0.106	−0.236 to 0.024	0.12	−0.12	−0.246 to 0.006	0.072
	β3	0.001	−0.020 to 0.021	0.933	0.007	−0.011 to 0.025	0.456
	β1+β3	0.005	−0.002 to 0.012	0.206	0.007	0.000 to 0.013	0.041
BBs	β0	0.91	0.830 to 0.989	<0.0001	1.083	0.973 to 1.193	<0.0001
	β1	−0.006	−0.016 to 0.005	0.286	−0.003	−0.017 to 0.011	0.707
	β2	−0.103	−0.175 to −0.031	0.009	−0.113	−0.222 to −0.004	0.051
	β3	0.006	−0.005 to 0.018	0.286	0.004	−0.011 to 0.020	0.566
	β1+β3	0.001	−0.003 to 0.004	0.743	0.002	−0.004 to 0.007	0.539
CCBs	β0	0.893	0.734 to 1.053	<0.0001	0.951	0.788 to 1.115	<0.0001
	β1	0.006	−0.015 to 0.027	0.597	0.004	−0.018 to 0.025	0.724
	β2	−0.12	−0.271 to 0.030	0.128	−0.142	−0.299 to 0.015	0.087
	β3	−0.005	−0.029 to 0.019	0.664	−0.001	−0.025 to 0.023	0.924
	β1+β3	0	−0.008 to 0.008	0.919	0.003	−0.006 to 0.011	0.518
Diuretics	β0	0.853	0.785 to 0.922	<0.0001	1.019	0.957 to 1.082	<0.0001
	β1	−0.008	−0.018 to 0.001	0.089	−0.022	−0.031 to −0.014	<0.0001
	β2	−0.031	−0.100 to 0.039	0.395	0.026	−0.047 to 0.099	0.492
	β3	0.007	−0.004 to 0.019	0.199	2.299	2.020 to 2.578	<0.0001
	β1+β3	−0.001	−0.005 to 0.003	0.65	−0.001	−0.037 to 0.036	0.964

β0: intercept value; β1: pretrend slope; β2: postinterruption immediate—level change; β3: postinterruption trend change; β1+β3: postinterruption—trend slope.

ACEiACE inhibitorARBsangiotensin receptor blockersBBsbeta blockersCCBscalcium channel blockers

**Table 3:** Single interrupted time-series analysis for cumulative incidence rate of any antihypertensive, ACEi, ARBs, BBs, CCBs and diuretics initiation before and after the pandemic onset by age groups in Sweden

Drug	Parameter	18–64 years			>65 years		
		Coefficient (%)	95% CI	P value	Coefficient (%)	95% CI	P value
Any	β0	1.834	1.602 to 2.066	<0.0001	5.41	4.738 to 6.082	<0.0001
	β1	0.014	−0.016 to 0.045	0.358	0.039	−0.048 to 0.126	0.384
	β2	−0.189	−0.415 to 0.038	0.113	−0.561	−1.221 to 0.099	0.106
	β3	0.006	−0.031 to 0.044	0.738	0.021	−0.088 to 0.130	0.708
	β1+β3	0.021	0.005 to 0.036	0.009	0.06	0.015 to 0.106	0.009
ACEi	β0	0.497	0.436 to 0.558	<0.0001	1.689	1.515 to 1.863	<0.0001
	β1	−0.002	−0.010 to 0.006	0.59	−0.03	−0.053 to −0.007	0.017
	β2	−0.049	−0.105 to 0.006	0.093	−0.236	−0.399 to −0.074	0.008
	β3	0.002	−0.007 to 0.011	0.7	0.026	0.002 to 0.051	0.044
	β1+β3	0	−0.004 to 0.003	0.792	−0.004	−0.011 to 0.004	0.331
ARBs	β0	0.649	0.536 to 0.762	<0.0001	2.273	1.947 to 2.598	<0.0001
	β1	0.007	−0.008 to 0.022	0.347	−0.015	−0.058 to 0.028	0.495
	β2	−0.051	−0.165 to 0.062	0.382	−0.411	−0.730 to −0.092	0.017
	β3	−0.001	−0.019 to 0.016	0.873	0.027	−0.019 to 0.072	0.26
	β1+β3	0.006	−0.001 to 0.012	0.086	0.012	−0.003 to 0.026	0.128
BBs	β0	0.895	0.817 to 0.973	<0.0001	2.317	2.086 to 2.548	<0.0001
	β1	0	−0.010 to 0.010	0.967	−0.023	−0.054 to 0.007	0.144
	β2	−0.066	−0.143 to 0.011	0.102	−0.32	−0.543 to −0.097	0.009*
	β3	0.001	−0.010 to 0.013	0.811	0.024	−0.008 to 0.055	0.15
	β1+β3	0.001	−0.003 to 0.006	0.602	0	−0.009 to 0.010	0.965
CCBs	β0	0.662	0.543 to 0.782	<0.0001	2.662	2.217 to 3.108	<0.0001
	β1	0.009	−0.007 to 0.025	0.262	−0.006	−0.065 to 0.053	0.85
	β2	−0.055	−0.171 to 0.061	0.363	−0.471	−0.908 to −0.034	0.043*
	β3	−0.008	−0.027 to 0.011	0.438	0.008	−0.054 to 0.071	0.793
	β1+β3	0.002	−0.006 to 0.009	0.674	0.003	−0.018 to 0.023	0.797
Diuretics	β0	0.541	0.508 to 0.574	<0.0001	3.063	2.845 to 3.282	<0.0001
	β1	−0.004	−0.008 to 0.001	0.094	−0.065	−0.095 to −0.034	<0.0001
	β2	0.028	−0.014 to 0.071	0.206	−0.106	−0.336 to 0.124	0.375
	β3	0.003	−0.003 to 0.009	0.308	0.058	0.024 to 0.092	0.002
	β1+β3	−0.001	−0.004 to 0.002	0.662	−0.007	−0.019 to 0.005	0.283

β0: intercept value; β1: pretrend slop; β2: postinterruption immediate—level change; β3: postinterruption trend change; β1+β3: postinterruption—trend slope.

ACEiACE inhibitorARBsangiotensin receptor blockersBBbeta blockerCCBscalcium channel blockers

Regarding initiation with specific antihypertensive classes, for ACEi, there was a significant (p=0.03) level change following the pandemic with rates dropping by −0.07% between the preinterruption and postinterruption period. This immediate level change was also observed after sex stratification (males (−0.07%), females (−0.09%)) (table 2, online supplemental figure 1,2). For patients older than 65 years, there was a significant negative prepandemic trend (p=0.02), with rates dropping by −0.03% per month. In this age category, an immediate decrease in level (β_2_=−0.24%, p=0.008), followed by a significant, positive change in slope (β_3_=+0.03%, p=0.044) was noted (table 3, online supplemental figure 4).

For ARBs, no statistically significant changes in relation to pandemic onset were detected prior to stratification (figure 1). However, in females, there was a significant postinterruption trend, with a +0.007% monthly increase (table 2, online supplemental figure 2). An abrupt decrease, indicated by a statistically significant level change, was detected in the patients >65 years, with no sustained effects (table 3, online supplemental figure 4).

For CCBs, no statistically significant differences were observed either before or after the age-stratified and sex-stratified analysis. Regarding initiation of BBs, there was a significant (p=0.03) level change following the pandemic with rates dropping by about 0.11% between the preinterruption and postinterruption periods, with no sustained effects. The same was observed for males (β_2_=−0.10%, p=0.03) (table 2, online supplemental figure 1), while this immediate decrease was even more apparent in patients older than 65 years (β_2_=−0.31%, p=0.009) (table 3, online supplemental figure 4). For diuretics, the results show a statistically significant decrease in the initiation during the preperiod (β_1_=−0.02%, p=0.002). At the time of interruption, there was no statistically significant change in level, but a positive change in slope was observed (β3=+0.013%, p=0.002). This may be related to the stronger significant change observed in patients older than 65 years. In this age category, prepandemic trend was negative, (p=0.02), with rates decreasing by −0.11% per month, and an immediate decrease in level (β2=−0.24%, p=0.008), followed by a significant, positive change in slope (β3=+0.06%, p=0.002).

## Discussion

In the present study, an immediate level change at the pandemic start interruption point was observed for all antihypertensive drugs. The trend change returned to the previous level of incidence during the following months after the early reduction, but the incidence remained lower from March 2020 to February 2021 compared with the same months in 2019. Two studies from the UK report similar findings. A cohort study on 618 161 people with type 2 diabetes in the UK found a 22% reduction in the rate of antihypertensives initiation between March and December 2020.^45^ Another study, based on monthly counts of medications dispensed, observed a decline in the dispensing of antihypertensive medications in the UK following the pandemic, with 491 306 fewer individuals than expected initiated on treatment.^46^ A Swedish study, which analysed weekly volumes of dispensed medication, found an increase in the observed versus predicted number of Defined Daily Doses (DDDs) for cardiovascular drugs in March 2020. The authors suggested stockpiling behaviour rather than an increase in the number of new users of medication as responsible for this change.^47^ This hypothesis is in line with the findings of our study, as we found a decrease in the new use of antihypertensives immediately after the pandemic started interruption point. Whether this drop may have long-term negative effects remains to be studied, which could prove difficult as consequences of missed treatment and risk factor control for CVDs do not become apparent for years.

The immediate reduction in all antihypertensives initiation in new users after the pandemic started was followed by the postinterruption trend reverting to positive, with an increase of 0.019% per month. A possible explanation for the increasing postinterruption trend could be catching up for the drop observed immediately after the onset of the pandemic, with no actual change in morbidity. Another potential explanation is an increase in CVD morbidity, as the COVID-19 pandemic may have negatively affected several CVD risk factors, including reports of a rise in blood pressure.^41^,^4348^ The COVID-19 infection also exacerbates CVD, and there is an increased burden of CVD in COVID-19 survivors.^7^,^9^ The increase in antihypertensives initiated was most pronounced in people over 65, and this patient population is known to be more susceptible to COVID-19 complications, including cardiovascular. The most commonly reported in-hospital cardiovascular complications of elderly COVID-19 patients were acute cardiac injury, arrhythmia and heart failure.^51 52^ Long-term complications include atrial fibrillation, myocardial ischaemia and pulmonary thromboembolism,^53^ at all ages, but the rate is notably higher in patients suffering from more severe disease, and old age in itself is a risk factor for severe COVID-19 infection. It has also been reported that COVID-19 may trigger new-onset high blood pressure in adults with pre-existing heart conditions or in older individuals.^54^ This increased incidence of hypertension in COVID-19 survivors is worrisome given the number of people affected by COVID-19.

Regarding specific classes, for CCBs, no statistically significant differences were observed. For BBs, there was immediate drop, but no significance in postpandemic slope or trend. For ACEi and diuretics, an increasing trend was found after the pandemic. As already mentioned, there were controversies early during the pandemic about possible negative effects of ACEis and ARBs in COVID-19 patients.^21 22^ It is a positive finding that the present study indicates no sustained negative effects on ACEi initiation in Sweden. The increase in ACEi use was most obvious in patients older than 65, where before the pandemic, there was a significant negative trend in ACEi use, and after the pandemic, there was a significant, positive change in slope. This population of patients older than 65 has a higher burden of comorbidities, which can explain these findings. ACEis are used for several CVDs, that is, after myocardial infarction, in heart failure and are recommended also to patients with Type 2 Diabetes Mellitus (T2DM) with hypertension due to positive effects on preserving kidney function. For diuretics, there was a positive change in slope, with trend changing from the prepandemic negative to postinterruption positive trends. Again, this was driven by changes observed in patients older than 65. Diuretics are rarely used as a monotherapy and are more commonly used in addition to ARBs, ACEis and BB.

No major sex differences were found, with similar patterns observed for both sexes. The only exception was for ARBs, where statistically significant changes were found only in females, with a significant postinterruption trend of a +0.007% monthly increase. The exact reasons for this finding are unclear.

This study has some limitations that need to be acknowledged. The study relies on prescription data from national health registers, which do not include clinical details such as diagnosis, blood pressure measurements or patient-reported outcomes. All data represent dispensed prescriptions and no information was available on prescriptions issued but not filled at pharmacies. There is also no information on if the patients actually used the medicines that were dispensed to them. The duration of preintervention and postintervention time periods was different, due to the time limitation in our drug data, which might have influenced the results. The data collection period might not have captured long-term effects, and future studies should consider a longer follow-up period to assess the sustained impact of the pandemic on antihypertensive drug initiation. Thus, due to the observational study design, we can only infer a causal correlation between the COVID-19 pandemic onset and the studied outcomes, and we cannot exclude the possibility of other coinciding factors driving the observed changes. However, the study used the preferred methodology for determining the effects of societal interventions and used data with complete population coverage.

## Conclusions

The present study found no major negative effects on COVID-19 pandemics on the initiation of antihypertensive drugs in Sweden. This is positive, considering the complicated interplay between CVDs and COVID-19 and the importance of continued provision of care for CVDs after the onset of pandemic. Lessons learnt from dealing with the COVID-19 pandemic could help healthcare planners develop strategies to ensure the management of chronic conditions during possible future crises.

## supplementary material

10.1136/bmjopen-2023-082209 online supplemental file 1

## Data Availability

Data are available on reasonable request.
