# Supplementary material for: Impact of the COVID-19 pandemic on initiation of antihypertensive drugs in Sweden: an interrupted time series study
Source: BMJ Open. 2024 Oct 16;14(10):e082209. doi: 10.1136/bmjopen-2023-082209 (PMC11487799; doi:10.1136/bmjopen-2023-082209)
Supplement: online supplemental file 1 [file bmjopen-14-10-s001.pdf]

Supplementary materials:

Supplementary table 1: Characteristics of antihypertensive medicine new users by types of the drugs, March 2019 to November 2021

	Duretics		Betablockers		CCBs		ACEis		ARBs	
Variables	Numbers	Percent	Numbers	Percent	Numbers	Percent	Numbers	Percent	Numbers	Percent
Total	136,987		139,168		86,176		140,141		315,362	
Sex										
Male	66,158	48.30	49,956	35.90	38,243	44.38	77,648	55.41	160,796	50.99
Female	70,829	51.70	89,212	64.10	47,933	55.62	62,493	44.59	154,566	49.01
Marital Status										
Married	65,973	48.26	56,778	40.88	40,347	46.94	67,250	48.11	159,866	50.78
Unmarried	136,712	51.74	82,098	59.12	45,607	53.06	72,544	51.89	154,939	49.22
Missing	275		292		222		347		557	
Educational level										
Primary school	32,192	24.04	22,153	16.50	18,752	22.30	33,808	24.86	67,445	21.76
Secondary school	62,184	46.45	54,494	40.58	38,303	45.56	62,853	46.22	145,289	46.88
Tertiary eductaion	39,508	29.51	57,637	42.92	27,023	32.14	39,315	28.91	97,152	31.35
Unknown	3,103		4,884		2,098		4,165		5,476	
Occupation status at the pandemic										
Employed	73,255	53.86	95,908	70.09	48,441	56.69	77,376	55.74	182,196	58.07
Unemployed/Retired	62,755	46.14	40,918	29.91	37,011	43.31	61,432	44.26	131,531	41.93
Missing	977		2,342		724		1,333		1,635	
Age at the first dispensing date of any hypertensive drug										
<18	393	0.29	3,288	2.36	328	0.38	511	0.36	279	0.09
18-64	67,084	48.97	102,181	73.42	45,750	53.09	73,294	52.30	160,172	50.79
≥65	69,510	50.74	33,699	24.21	40,098	46.53	66,336	47.34	154,911	49.12
Country of birth										
Sweden	110,265	80.50	112,152	80.59	68,200	79.15	10,7631	76.81	258,553	81.99
Nordics excluding Sweden	6,044	4.41	3,474	2.50	3,584	4.16	5,841	4.17	13,331	4.23

Eu28 except the Nordics	5,326	3.89	5,225	3.75	3,452	4.01	6,291	4.49	10,859	3.44
Out of Europe	15,344	11.20	18,310	13.16	10,934	12.69	20,367	14.53	32,603	10.34
Missing	8		7		6		11		16	

Supplementary table 2: Difference in monthly incidence relative to the same month in 2019

Month	Any anti-HTN	ACEi	ARBs	BBs	CCBs	Diuretics
Mar-20	-0.04	-0.11	0.00	-0.13	-0.01	-0.07
Apr-20	-0.36	-0.21	-0.16	-0.22	-0.14	-0.19
May-20	-0.54	-0.24	-0.24	-0.26	-0.23	-0.28
Jun-20	-0.05	-0.11	-0.03	-0.07	-0.07	-0.04
Jul-20	-0.13	-0.08	-0.07	-0.09	-0.07	-0.14
Aug-20	-0.22	-0.11	-0.08	-0.14	-0.1	-0.12
Sep-20	0.00	-0.09	0.01	-0.06	-0.03	-0.05
Oct-20	-0.12	-0.11	-0.04	-0.12	-0.08	-0.08
Nov-20	-0.20	-0.12	-0.06	-0.15	-0.09	-0.11
Dec-20	-0.07	-0.09	-0.03	-0.08	-0.08	-0.08
Jan-21	-0.32	-0.14	-0.13	-0.16	-0.18	-0.18
Feb-21	-0.10	-0.09	-0.08	-0.05	-0.09	-0.08
Mar-21	0.12	-0.15	0.05	-0.12	-0.03	-0.08
Apr-21	-0.04	-0.17	-0.04	-0.15	-0.10	-0.14
May-21	-0.10	-0.19	-0.08	-0.16	-0.10	-0.21
Jun-21	0.33	-0.10	0.16	0.00	0.04	-0.02
Jul-21	-0.08	-0.13	-0.05	-0.13	-0.13	-0.11
Aug-21	-0.04	-0.12	-0.01	-0.13	-0.05	-0.15
Sep-21	0.22	-0.11	0.08	-0.07	-0.01	-0.04
Oct-21	0.10	-0.15	-0.01	-0.15	-0.07	-0.14
Nov-21	0.27	-0.12	0.01	-0.12	-0.04	-0.08

Supplementary Figure 1. Cumulative monthly incidence of initiation of antihypertensives drugs in males, Sweden, March 2019 to November 2021

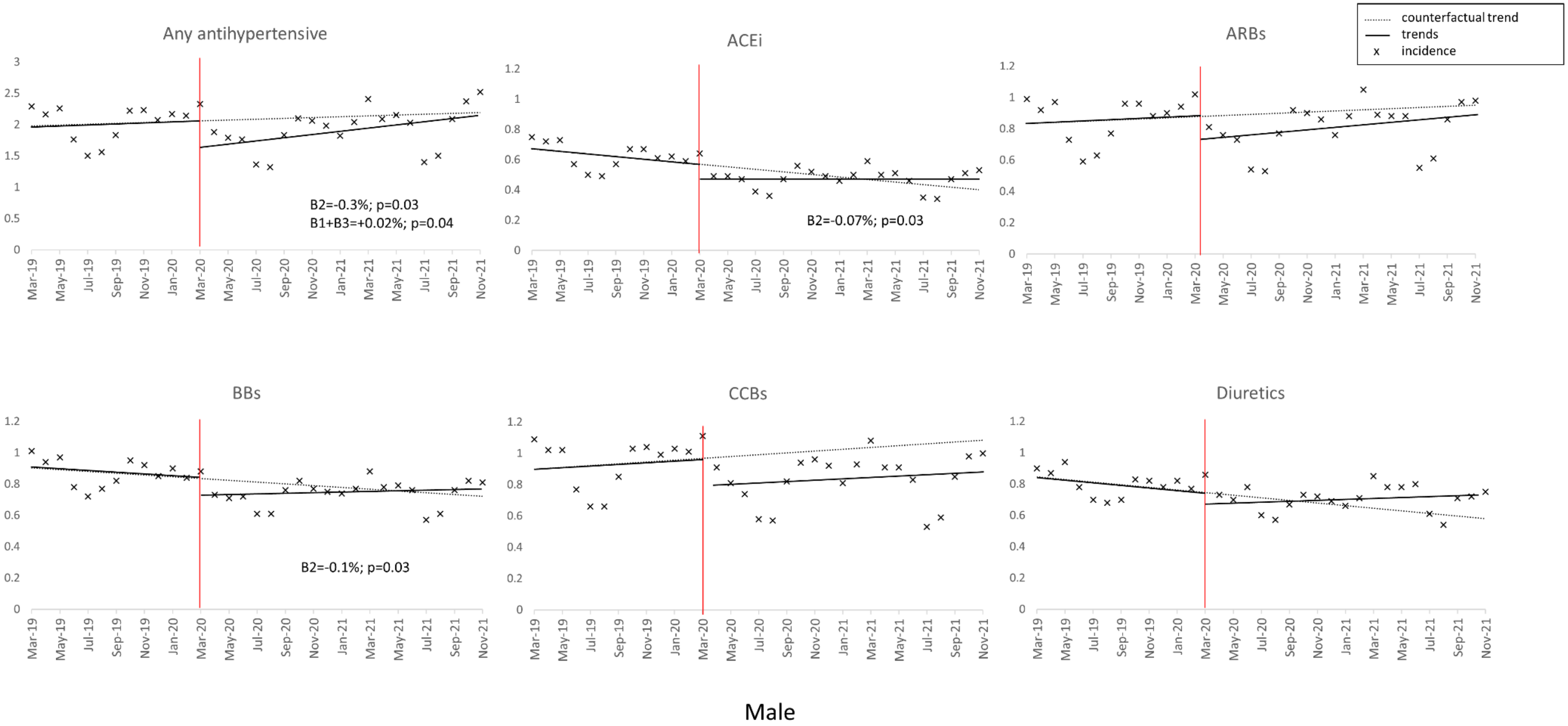

Supplementary Figure 2. Cumulative monthly incidence of initiation of antihypertensives drugs in females, Sweden, March 2019 to November 2021

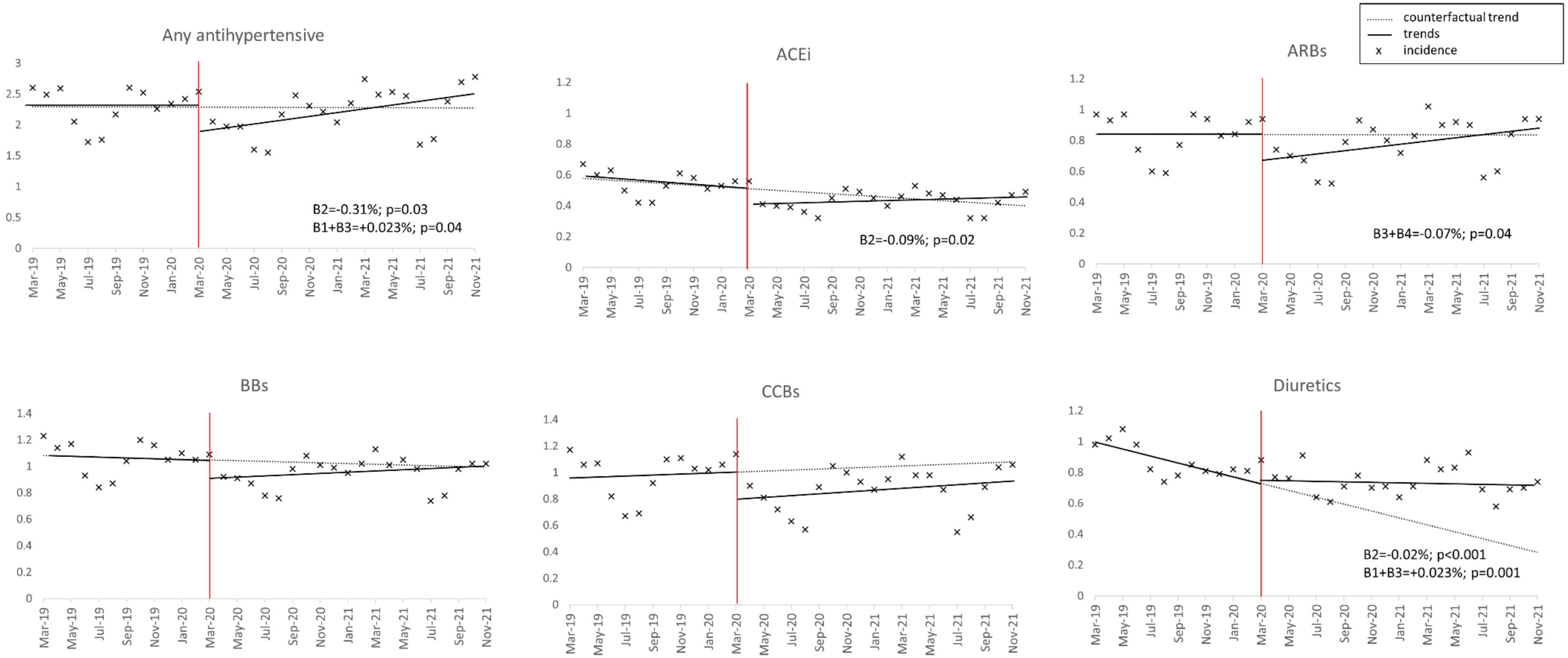

Female

Supplementary Figure 3. Cumulative monthly incidence of initiation of antihypertensives drugs in age group 18-64 years, Sweden, March 2019 to November 2021

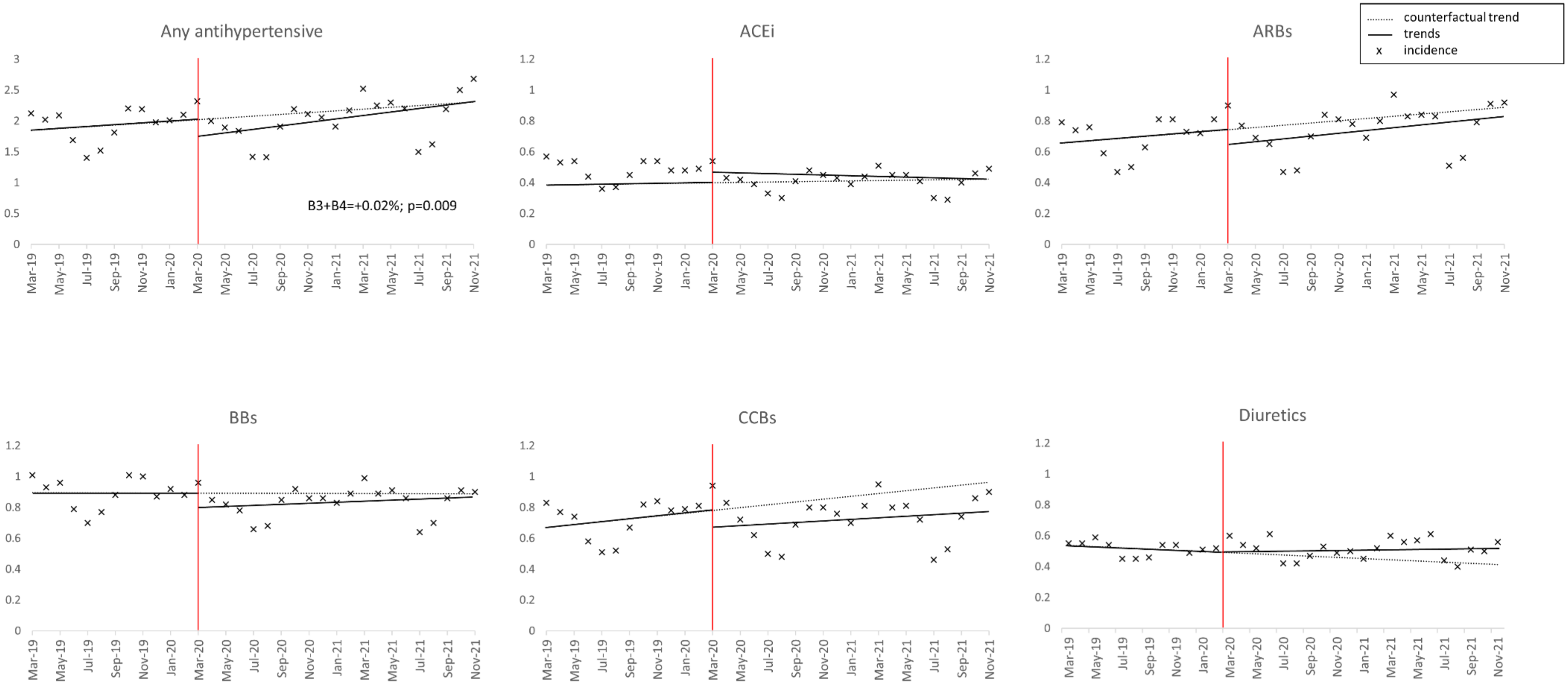

18-64 years

Supplementary Figure 4. Cumulative monthly incidence of initiation of antihypertensives drugs in age group >65 years, Sweden, March 2019 to November 2021

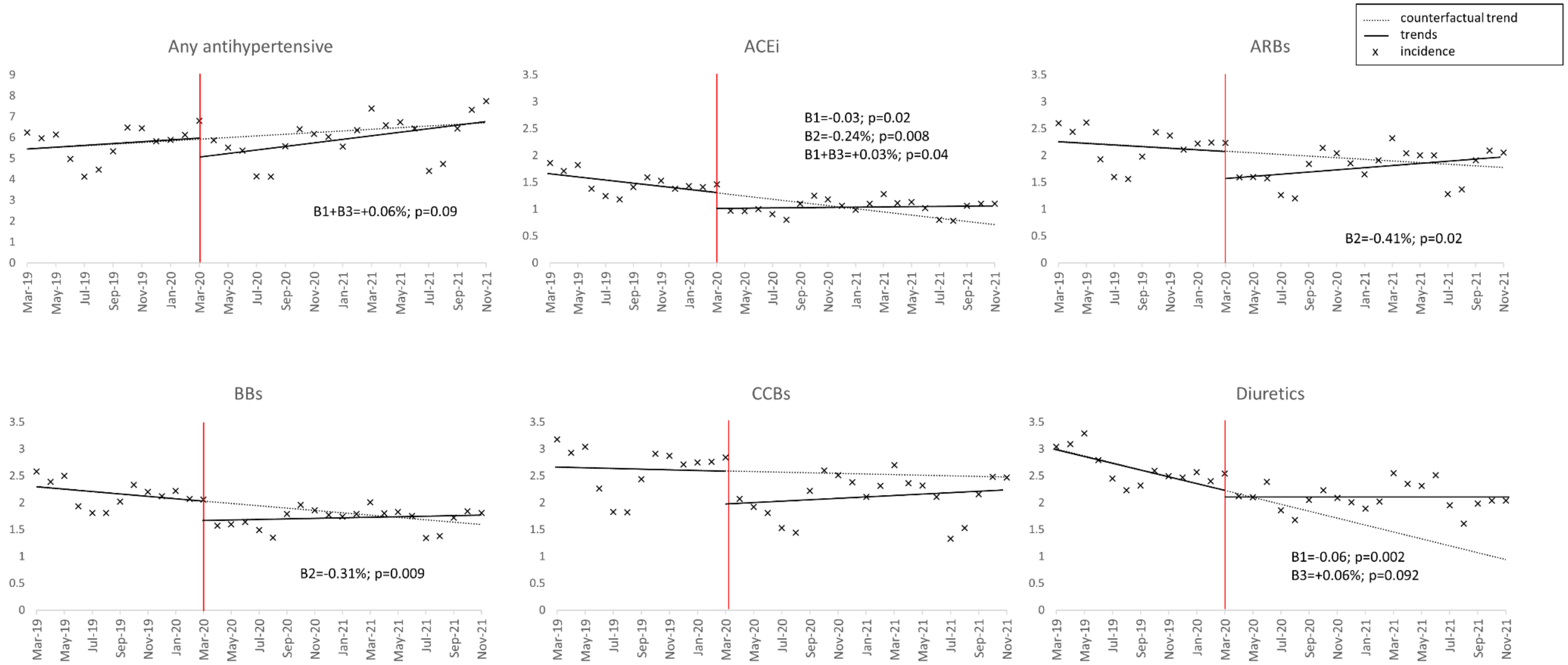

>65 years
